# Comprehensive evaluation model for health grade of multi-component compound release materials based on fuzzy comprehensive evaluation with grey relational analysis

**DOI:** 10.1038/s41598-022-23887-2

**Published:** 2022-11-17

**Authors:** Qifan Wang, Jianhui Du, Jun Shen, Yiming Wang

**Affiliations:** 1 College of Material Science and Engineering, Northeast Forestry University, Harbin, 150040 China; 2 Department of Building Science, Tsinghua University, Beijing, 100084 China; 3 Beijing Baidu Netcom Science and Technology Co., Ltd, Beijing, 100085 China

**Keywords:** Health care, Health occupations, Risk factors, Materials science

## Abstract

The selection of materials is a complex and elusive process due to the multiple factors related to health. For the effective selection of materials, a comprehensive model for evaluating the health grade of materials was proposed with the mathematical method of the fuzzy comprehensive evaluation (FCE) and grey relational analysis (GRA). A health evaluation procedure scheme of the AgBB as well as standards of the forestry industry of the People’s Republic of China and standards of the health industry of the People’s Republic of China were referred to during the process. Based on Qt technology, a computer program was developed to calculate the compound comprehensive evaluation models. The data of *Choerospondias axillaris* with different lacquers were used as practical examples firstly, and then, the emission and odor characteristics of *Xylosma racemosum* (Sieb. et Zucc.) Miq. with PU, UV, and waterborne coating were explored with the technology of GC–MS/O. Results showed that: a total of 21 odorants were identified from *Xylosma racemosum* with PU, UV, and waterborne coating. The health grade of three *Xylosma racemosum* samples was UV > waterborne > PU, with the health grade of UV coating being “A”, and the boards with PU and the waterborne coating were “N”, with a fuzzy comprehensive index of *P*_PU_ = 0.5151, *P*_waterborne coatings=_0.4950. Based on the results, it is proved that the establishment of the comprehensive evaluation model based on FCE and GRA successfully converts qualitative evaluation into quantitative evaluation; in addition, effectively evaluates the health grade of materials. This study provides a better understanding of materials’ health grades, which will aid consumers in reducing indoor air pollution and using their products properly.

## Introduction

People’s demands for a better quality of life have led to more research into the release of volatile organic compounds (VOCs) from wooden boards, which are popular decorative materials. Though numerous studies have been done on VOC and odor release from wooden boards with different decorative materials^1–7^ by developed technology and method^8,9^, people still find it very difficult to choose panels in daily life. Traditionally, people evaluated the health effects of products based on their concentration. However, the health grade of products with the same concentration may differ due to the different toxicity of compound monomers and odor characteristics. As a consequence, people become increasingly aware of the limitations of only considering the material’s health grade from a concentration standpoint.

Several studies have been undertaken in the field of selecting wooden materials with regard to health performance. Jiang et al*.*^10^ studied the effects of VOCs from different decorative particleboards on indoor air quality by the comprehensive index evaluation method^11^, which considers the largest single-factor index and the arithmetic average index, and found the VOCs from the PVC-faced particleboard had the lowest impact on indoor air quality. Li et al*.*^12^ analyzed the carcinogenic hazard of benzene series (including benzene, toluene, ethylbenzene, p-xylene, and styrene) from particleboard with different lacquered decorations. The health risk model of US EPA^13^, which focused on the properties of key compounds, was used to evaluate the boards. Although there is more and more attention to specific component concentration or the risk of monomer compound, the evaluation considering multi factors has not been realized, and problem such as odor is always ignored. The health risks of VOCs and odor from PVC-overlaid particleboard were investigated by Wang et al*.*^8^ by the synthetic index-olfactory method. This study brought the sensory problem into view, but it still failed to achieve the quantitative evaluation. The health-related evaluation procedure scheme of AgBB, which had a high reference value, well-considered the effect of the various indices on the health grade of the product^14^. However, the procedure only determined whether the materials are suitable for the application of indoor use. The health grade of material is unobtainable and it is unable to distinguish the health grade of different materials. Related research^15^ tried to evaluate the *Choerospondiasaxillaris* (Roxb.) Burtt et Hill with different lacquer treatments referring to the procedure scheme of AgBB. Based on the subjective comparison, the wood with waterborne coating proved to be the best choice among these three boards in this study, but it also pointed out that there was a problem that the concentration of non-assessable compounds within the board with waterborne coating was higher than those with UV and PU coatings. Therefore, to achieve material rating and horizontal comparison, it is necessary to explore a method to put several affecting factors, such as concentration, toxicity, and sensory properties into consideration. The effects of compounds with low molecular weight should be considered at the same time.

The selection of materials in engineering design is a complex and elusive process that takes into account multiple factors, so multiple scientific methods have been employed to validate the selection. Several researchers have proposed a hybrid TOPSIS-PSI method for effective material selection in marine applications. Based on a logical combination of PSI and TOPSIS algorithms, a selection index value has been calculated and ranked in ascending or descending order^16,15^. Additionally, a hybrid fuzzy Preference Selection Method (f-PSI) was developed to select an optimal alternative ship body material based on the physical, mechanical, and corrosion characteristics of a novel hybrid aluminum metal alloy composite^17^. A fuzzy logic-based model was also used to predict the effect of surface reconstruction parameters on the response of surface deviation and the final memory space required after scanning the transtibial prosthesis socket^18^. Along with the aforementioned methods, artificial neural networks (ANNs) are also widely used to advance prediction models in several fields^19–21^.

Following consideration of the actual situation and multiple criteria, a fuzzy comprehensive evaluation, which is derived from fuzzy mathematics theory, was selected. By using fuzzy mathematics and the concept of fuzzy relationship synthesis, it was able to quantify indicators with unclear boundaries. The concept of “fuzzy set” was first proposed by Professor L.A. Zadeh in 1965^22^, which broke through Cantor's “classical sets theory” and laid the foundation of “fuzzy set theory”. The means of fuzzy mathematics can expand the application scope of mathematics from the field of accurate phenomena to the field of fuzzy phenomena, it can deal with complex system problems by using accurate mathematical methods. The basic idea of the fuzzy logic-based model is to use the degree of belonging instead of belonging or not belonging and obtain an accurate quantitative value. According to the “membership grade theory” of fuzzy mathematics, the qualitative evaluation can be transformed into quantitative evaluation, and the comprehensive evaluation results considering multi factors will be obtained in this process. At the same time, the grey relational analysis method, which is based on the degree of similarity or difference between the development trends of indicators was also used. It is a method to measure the degree of correlation between factors, and can quantitatively describe and compare the development and change trend of a system. The basic idea is to judge whether the relation is close by determining the geometric similarity of the reference data column and several comparison data columns.

The study proposed a comprehensive evaluation method based on relevant standards and experiments. Health grade models for multicomponent compound release materials were constructed using fuzzy comprehensive evaluation (FCE) and gray relationship analysis (GRA). The corresponding computer software was programmed for realizing the rapid calculation of a comprehensive evaluation model. The health grade of two kinds of wood with different lacquer treatments on emissions was evaluated by the way of quantitative expression. At the same time, the emission, including VOCs and VVOCs and odorants of *Xylosma racemosum* (Sieb. et Zucc.) Miq. with different lacquer treatments were investigated.

## Theoretical background

### LCI value

The LCI value (Least Concentration of Interest) reflects a measure of toxicological evaluation of substances that is set below which, according to the most reasonable professional judgment and based on concentration levels, it would not be expected to produce adverse effects. The concept originated from European scientists and was first published in the ECA report in 1997^23^. It was then adopted and developed by the scheme of the Committee for Health-related Evaluation of Building Products (Ausschuss für die gesundheitliche Bewertung von Bauprodukten, AgBB) and French environmental and occupational health and Safety Agency (AFSSET) (now called French Food Safety Agency, ANSES).

The LCI value is derived from air quality guidelines (AQG) or occupational exposure limits (OELVs). When VOCs have been assessed by national or international committees, the lowest value is used. Additionally, if no evaluation of the compound has been conducted through the above procedures, the TLV value of the American Conference of Governmental Industrial Hygienists (ACGIH), the MAK value published by the German Research Foundation (DFG) or the WEEL value of the American Industrial Hygiene Association (AIHA), and some other minimum exposure value of the compound in the workplace determined by other countries also can be used as the LCI value^24^. For compounds whose LCI values have not been determined even by the above methods, toxicity assessments may rely on predictive approaches, such as read-across and grouping of substances. For some compounds, the LCI cannot be determined even with the read-across method. In this case, the total concentration of “not assessable” compounds will be calculated and evaluated to avoid overestimating the level of non-assessable substances emitted by a product.

### Health-related evaluation procedure of AgBB

AgBB built the health-related evaluation procedure scheme for volatile organic compounds^14^. The substances whose concentrations in the test chamber air are equal to or greater than 5 µg/m^3^ will be considered. Based on Report 19 of the European Collaborative Action on Indoor Air Quality and its Impact on Man^26^, the total concentration of VOCs (all individual substances within the retention range C_6_–C_16_) was calculated and expressed as TVOC. The total concentration of SVOC (all individual substances within the retention range > C_16_–C_22_) was calculated and expressed as TSVOC. VVOCs represent all individual substances within the retention range < C_6._

For the evaluation of each compound *i*, the ratio *R*_*i*_ is established as defined in Eq. (1)^28^:

1 $$R_{{\text{i}}} \, = \,C_{{\text{i}}} /{\text{LCI}}_{{\text{i}}} ,$$

where *C*_*i*_ is the chamber concentration of compound*i*. For *R*_*i*_ < 1, it is assumed that there will be no effects. If several compounds with a concentration > 5 µg/m^3^ are detected, additive effects are assumed, and then *R* was gotten used in the Eq. (2).

2 $$R\, = \,{\text{sum of all}}\, = \,{\text{sum all ratios}}\,\left( {R_{{\text{i}}} C_{{\text{i}}} /{\text{LCI}}_{{\text{i}}} } \right).$$

A variety of factors are taken into account, including TVOC, TSOC, the concentration of carcinogenic substances, R-value, the total concentration of “not assessable” compounds, as well as sensory evaluations, the AgBB evaluation procedures stipulate that the products should be checked after 3 days, TVOC and emissions of carcinogenic substances in EU categories 1A and 1B are assessed, and then, the product with TVOC ≤ 10,000 μg⋅m^−3^ and carcinogens of EU cat. 1A and 1B ≤ 10 μg⋅m^−3^ will be evaluated after 28 days, or else the product will be rejected. The product suitable for indoor use should meet the following requirements after 28 days successively: (1) TVOC ≤ 1000 μg⋅m^−3^. (2) TSVOC ≤ 100 μg⋅m^−3^. (3) Carcinogens of EU cat. 1A and 1B ≤ 1 μg⋅m^−3^. (4) R ≤ 1. (5) The total concentration of “not assessable” compounds” ≤ 100 μg⋅m^−3^. Sensory testing, voluntarily, also be added to the evaluation procedure. If an odor intensity, determined by a trained panel^29^, not exceeded 7 pi, the sensory testing is considered passed.

## Model implementation

### Theoretical basis

**Figure 1:**
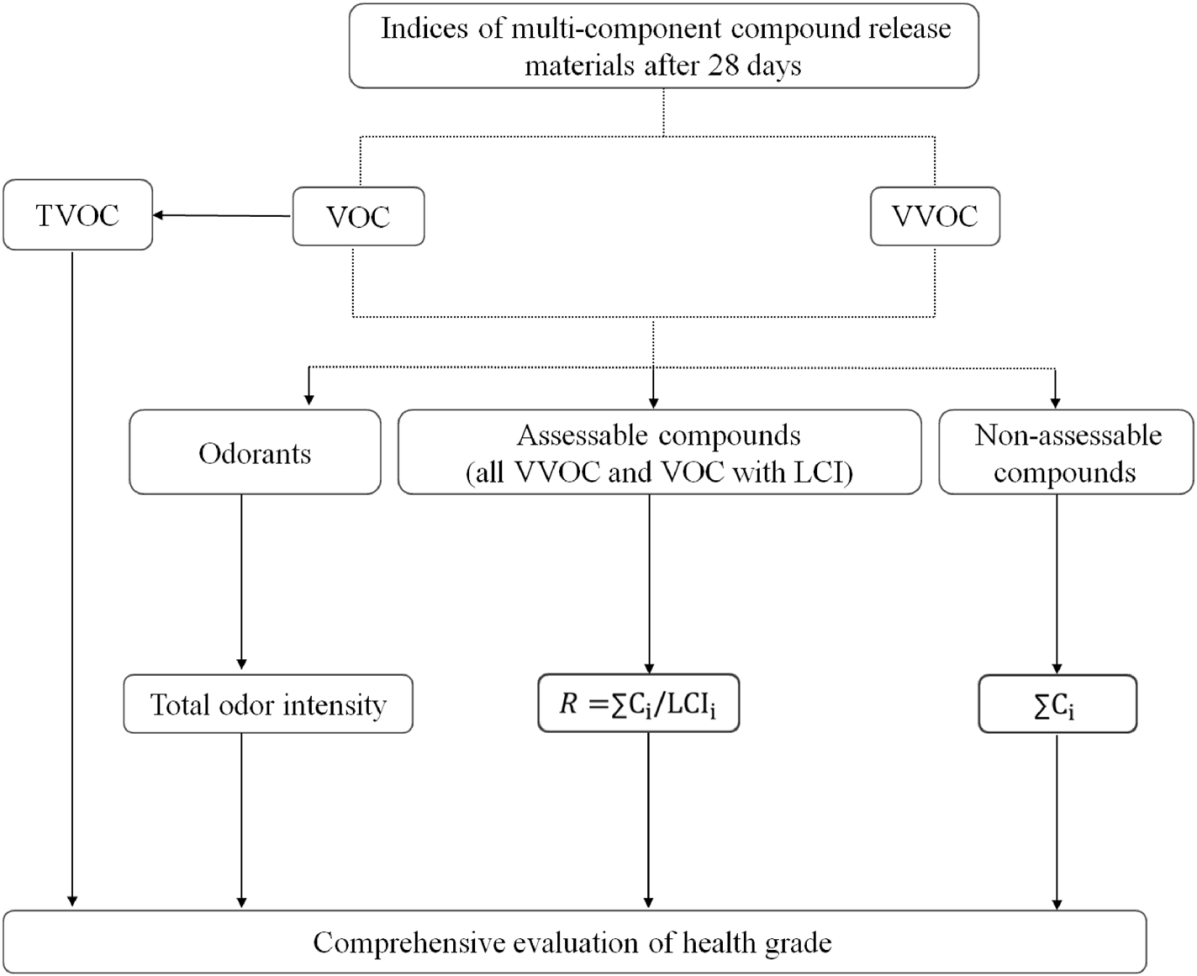
A comprehensive evaluation of the health level of multi-component materials.

The proposed model is based on the above evaluation procedure of AgBB, focusing mainly on TVOC, total odor intensity, *R*, and the concentration of non-assessable compounds. The evaluation process for the health level of multi-component emission materials was shown in Fig. 1, which mainly focuses on the characteristics of each index after 28 days. The evaluation first pays attention to TVOC, at the same time, the compounds which belong to VVOCs were also considered. The evaluation consists of subjective evaluation and objective evaluation, mainly including (1) TVOC, *R*, and the concentration of non-assessable compounds. (2) Odor evaluation based on total odor intensity (refer to Japanese standards^30^). Due to the complex composition of odor substances, different compounds can affect each other. Since multiple odorous compounds interact in a complex way, an odor’s total odor intensity is determined by the fusion effect.

In the comprehensive evaluation model, different indices are considered, including subjective and objective measures. The health grade is gotten by FCE with GRA. Meanwhile, the model also gives attention to the limit value of the single index, referring to the evaluation procedure of AgBB and the forestry industry standard of the People’s Republic of China^31^.

### Term weighting with grey relational analysis method

In the evaluation process, the weight corresponds to the relative importance of each factor to the evaluation objectives and has an impact on the evaluation outcomes. Therefore, the GRA (gray relational analysis) was used to determine the weight of the impact evaluation results of various indicators.

To estimate the correlation degree of the impact of different indicators on health, relevant test data were selected, and the weights of different indicators were comprehensively examined using grey relational analysis. The weight analysis indexes used in this method include the R-value, the total concentration of VOCs, the concentration of non-assessable compounds, and the total odor intensity. Based on relevant standards, the comparison sequence (subsequence) of indicators affecting the behavior of the system was obtained through expert evaluation. Based on the absolute degree of the index standard, an original relative rating of the sample’s health was determined. On this basis, the influence trend variables may be determined through time, environmental factors, and painted wood with known health grades. Through horizontal and vertical comparative analysis, the health rating of the board was corrected, and the reference sequence (general target) reflecting the behavior characteristics of the system was obtained; the specific steps were as follows.

#### Determination of the analysis sequence

Based on relevant standards, the comparison sequence that affects system behavior was gotten through expert evaluation, which was composed of the score value of each indicator. The comparison sequence (subsequence) was represented as the Eq. (3):

3 $${{X}_{i}= X}_{i}\left(k\right)\left|k=1,\hspace{0.33em}2,\hspace{0.33em}\cdots ,\hspace{0.33em}n\right., i=\mathrm{1,2},\cdots ,m$$

The analytic index system was set according to the analysis purpose, and the analysis data was collected.

If n data sequences form the following matrix, Eq. (4) was gotten:

4 $$\left({X}_{1}^{^{\prime}},{X}_{2}^{^{\prime}},\cdots ,{X}_{n}^{^{\prime}}\right)=\left(\begin{array}{cccc}{x}_{1}^{^{\prime}}(1)& {x}_{2}^{^{\prime}}(1)& \cdots & {x}_{n}^{^{\prime}}(1)\\ {x}_{1}^{^{\prime}}(2)& {x}_{2}^{^{\prime}}(2)& \cdots & {x}_{n}^{^{\prime}}(2)\\ \vdots & \vdots & \ddots & \vdots \\ {x}_{1}^{^{\prime}}(m)& {x}_{2}^{^{\prime}}(m)& \cdots & {x}_{n}^{^{\prime}}(m)\end{array}\right)$$

If m is the number of indicators, the Eq. (5) was showed as follows:

5 $${X}_{i}^{^{\prime}}=({x}_{i}^{^{\prime}}(1),{x}_{i}^{^{\prime}}(2),\cdots ,{x}_{i}^{^{\prime}}(m){)}^{T},\hspace{0.33em}\hspace{0.33em}\hspace{0.33em}\hspace{0.33em}i=\mathrm{1,2},\cdots ,n$$

Based on the absolute degree of the indicator’s standard, the original relative grade of the health of the product was obtained through the certainty trend of some factors, such as time and environmental factors. The reference sequence (general target) that reflects system behavior was gotten with the grade correction by horizontal and vertical comparative analysis, which showed in Eq. (6).

6 $$Y=Y(k)\left|k=\mathrm{1,2},\cdots ,n\right.$$

The reference sequence was an ideal comparison standard, which can be formed by optimal value (or the worst value) of each index, or select other reference values according to the evaluation purpose (Eq. (7)).

7 $${X}_{0}^{^{\prime}}=\left[{x}_{0}^{^{\prime}}(1),{x}_{0}^{^{\prime}}(2),\cdots ,{x}_{0}^{^{\prime}}(m)\right]$$

#### Data dimensionless

To eliminate the adverse effects of different dimensions of data in each index column on the comparison results in the system, the initial value method (Eq. (8)) was used to get the dimensionless index.

8 $${X}_{i}(k)=\frac{{x}_{i}^{^{\prime}}(k)}{{x}_{i}^{^{\prime}}(1)}$$

In the equation, $i=0, 1, 2,...n; k=1, 2,...m$.

The following matrix (Eq. (9)) was formed by a dimensionless data sequence.

9 $$\left({X}_{0},{X}_{1},\cdots ,{X}_{n}\right)=\left(\begin{array}{cccc}{x}_{0}(1)& {x}_{1}(1)& \cdots & {x}_{n}(1)\\ {x}_{0}(2)& {x}_{1}(2)& \cdots & {x}_{n}(2)\\ \vdots & \vdots & \ddots & \vdots \\ {x}_{0}(m)& {x}_{1}(m)& \cdots & {x}_{n}(m)\end{array}\right)$$

#### Calculation of absolute difference and correlation coefficient

Then, the absolute difference between the comparison sequence and reference sequence was determined.

The correlation coefficient of each parameter in each comparison sequence and the corresponding parameter of the reference sequence was calculated by the Eq. (10).

10 $${\zeta }_{i}(k)=\frac{\underset{i}{min }\underset{k}{min}\left|{x}_{0}(k)-{x}_{i}(k)\right|+\rho \cdot \underset{i}{max}\underset{k}{max}\left|{x}_{0}(k)-{x}_{i}(k)\right|}{\left|{x}_{0}(k)-{x}_{i}(k)\right|+\rho \cdot \underset{i}{max}\underset{k}{max}\left|{x}_{0}(k)-{x}_{i}(k)\right|}$$

In the equation, $k=1, 2,...m$, *ρ* is the distinguishing coefficient, which normally takes ρ  = 0.5.

#### Calculation of correlation sequence

The correlation coefficient was the value of the correlation degree between the comparison sequence and the reference sequence. For the information was too scattered to facilitate the overall comparison, it was necessary to concentrate the correlation coefficients into one value, and calculate the mean value of the correlation coefficients of the corresponding indicators of the comparison sequence and the reference sequence respectively, which was called the correlation sequence and recorded as Eq. (11).

11 $${r}_{0i}=\frac{1}{m}{\sum }_{k=1}^{m}{\zeta }_{i}(k)$$

The correlation coefficient is normalized to obtain the weight proportion of each comparison sequence to the reference sequence. The weight of indicator i was calculated by Eq. (12).

12 $${\mathrm{W}}_{\mathrm{i}}={r}_{0i}\sum_{k=1}^{m}{r}_{0k}$$

Through the calculation of Matlab, the weight set was gotten in the Eq. (13).

13 $$\mathrm{W}=\left\{0.2790, 0.2664, 0.2122, 0.2424\right\}$$

Therefore the weight of the R-value, the total concentration of VOCs, the concentration of non-assessable compounds, and the total odor intensity were 0.2790, 0.2664, 0.2122, 0.2424, respectively.

### Proposed fuzzy comprehensive evaluation method

This mathematical model for the comprehensive health evaluation of multi-component compound release materials is based on the principles of the FCE mathematical model, combined with the health evaluation procedure established by the AgBB, the relevant standards of the forestry industry of the People’s Republic of China and the standards of the health industry of the People’s Republic of China^32^. The steps were as follows:

#### Determination of the indicator set (*U*)

Based on the “Health-Related Evaluation Procedure for Volatile Organic Compounds (VVOCs, VOCs, and SVOCs) Emissions from Building Products”, the indicator set was determined by the emission characteristics of the material after 28 days, with the value of TVOC, R, the concentration of non-assessable compounds, and total odor intensity. The indicator set was shown in Eq. (14)

14 $$U=\left\{{u}_{i}\right\}=\left\{{u}_{1},{u}_{2},{u}_{3},{u}_{4}\right\}=\left\{R ,\mathrm{TVOC},{\sum \mathrm{C}}_{\mathrm{non}-\mathrm{assessable}},\mathrm{ total odor intensity}\right\}$$

#### Establishment of comment set (*V*)

The comment set, shown in Eq. (15), was the overall evaluation, based on different indicators, for the health grade of materials.

15 $$V=\left\{{v}_{i}\right\}=\left\{{v}_{1},{v}_{2},{v}_{3},{v}_{4},{v}_{5}\right\}=\left\{\mathrm{A},\mathrm{B},\mathrm{C},\mathrm{Q},\mathrm{ N}\right\}.$$

In this evaluation system, the grade of A. B, C, Q, and N represented “excellent”, “good”, “medium”, “qualified” and “unqualified” respectively. From A to N, the health level decreased in turn, while the grade “Q” equaled to the level of “produce is suitable for indoor use”, and the grade “N” equaled to the level of “reject” in the procedure of AgBB.

#### Calculation of quality index (*I*_*i*_)

Since all indicators in this model were contrary indicators, the direction of evaluation indicators did not need to be unified for the contrary of all indicators in this model. The optimal limit of each index in the model was 0. Considering the actual situation of materials, the corresponding relationship was shown in Eq. (16):

16 $${C}_{i}={X}_{i}$$

where *C*_*i*_ is the index value of i after direction unification, and *X*_*i*_ is the representative value of the indicator.

To integrate the measurement, the *C*_*i*_ was converted to quality index, which can be calculated with the following equation:

17 $${I}_{i}={I}_{jmin}+\frac{{C}_{i}-{S}_{ij(1)}}{{S}_{ij\left(2\right)}-{S}_{ij(1)}}$$

where *I*_*i*_ is the quality index of index *i*, *I*_*jmin*_ is the minimum value of index *i* index under the grade of *j, S*_*ij*(1)_ and *S*_*ij*(2)_ were the lower limit and upper limit under the grade of *j.*

According to the comment set, the m value, representing the number of grades is 5, and the minimum value of the quality index for index *i* at grade *j, I*_*1min*_, *I*_*2min*_, *I*_*3min*_, *I*_*4min*_, *I*_*5min,*_ were 0, 1.0, 2.0, 3.0, and 4.0 respectively. The grade limit value of each index (Table 1) was divided according to the evaluation procedure of AgBB and the forestry industry standard of the People’s Republic of China. The equal allocation was used to divide the unspecified grade.

**Table 1:** Limit value of membership function of each index.

Index and grade	A	B	C	Q	N
R	≤ 0.25	≤ 0.5	≤ 0.75	≤ 1	> 1
TVOC	≤ 220	≤ 500	≤ 750	≤ 1000	> 1000
${\sum \mathrm{C}}_{\mathrm{non}-\mathrm{assessable}}$	≤ 25	≤ 50	≤ 75	≤ 100	> 100
$\mathrm{total odor intensity}$	≤ 8	≤ 15	≤ 20	≤ 25	> 25

#### Establishment of membership judgment matrix (*R*)

The membership degree is the most basic and important concept in fuzzy evaluation. For an indicator in the indicator set, the purpose was to obtain the membership of the research object relative to each comment in the comment set. The U × V membership judgment matrix of *R* was established in this part, and the assignment method was used to determine the membership function^33,34^. According to the characteristics of model data, and referring to relevant standards of the health industry of the People's Republic of China, the membership function of half trapezoid and triangle were chosen to calculate the membership degree *r*_ij._. The model consisted of 5 piecewise functions, and the related subordinate function is shown in the following equation.

when *j* = 1,

$${r}_{ij}=\left\{\begin{array}{c}1.0\hspace{0.33em}\hspace{0.33em}\hspace{0.33em}\hspace{0.33em}\hspace{0.33em}\hspace{0.33em}\hspace{0.33em}\hspace{0.33em}\hspace{0.33em}\hspace{0.33em}\hspace{0.33em}{I}_{i}\in \left[0, 1.0\right]\\ \frac{m-{I}_{i}}{m-1.0}\hspace{0.33em}\hspace{0.33em}\hspace{0.33em}\hspace{0.33em}{I}_{i}\in \left(1.0, \right.\left. m\right]\end{array}\right.$$

when *j* = 2,3,…*m*–1

18 $${r}_{ij}=\left\{\begin{array}{c}\frac{{I}_{i}-(j-3m+2.5)}{3m-2.5}\hspace{0.33em}\hspace{0.33em}\hspace{0.33em}\hspace{0.33em}{I}_{i}\in \left[j-3m+2.5, j\right]\\ \frac{j+m-1-{I}_{i}}{m-1.0}\hspace{0.33em}\hspace{0.33em}\hspace{0.33em}\hspace{0.33em}\hspace{0.33em}\hspace{0.33em}\hspace{0.33em}\hspace{0.33em}\hspace{0.33em}\hspace{0.33em}\hspace{0.33em}{I}_{i}\in \left(j, \right.\left. j+m-1\right]\end{array}\right.$$

when *j* = *m*,

$${r}_{ij}=\left\{\begin{array}{c}\frac{{I}_{i}+2m-2.5}{3m-2.5}\hspace{0.33em}\hspace{0.33em}\hspace{0.33em}\hspace{0.33em}{I}_{i}\in \left[-2m+2.5,m\right]\\ 1.0\hspace{0.33em}\hspace{0.33em}\hspace{0.33em}\hspace{0.33em}\hspace{0.33em}\hspace{0.33em}\hspace{0.33em}\hspace{0.33em}\hspace{0.33em}\hspace{0.33em}\hspace{0.33em}\hspace{0.33em}\hspace{0.33em}\hspace{0.33em}\hspace{0.33em}\hspace{0.33em}\hspace{0.33em}\hspace{0.33em}\hspace{0.33em}{I}_{i}\in \left(m, \right. \left.\infty \right)\end{array}\right.$$

In the equation, *r*_*ij*_ is the degree of possibility, for a product, the indictor *u*_*i*_ was devided to *v*_*j*_ comment, which represents the membership of *i* to *j. I*_*i*_ was the quality index, *m* was the number of grades (*m* = 5 in this model). The Eq. (19) was obtained after bringing into the Eq. (18).

$${r}_{1}=1.0 {I}_{i}\le 1.0$$

$${r}_{1}=(5.0-{I}_{i})/4.0 {I}_{i}>1.0$$

$${r}_{2}=({I}_{i}+10.5)/12.5 {I}_{i}\le 2.0$$

$${r}_{2}=(6.0{-I}_{i})/4.0 {I}_{i}>2.0$$

$${r}_{3}=({I}_{i}+9.5)/12.5 {I}_{i}\le 3.0$$

$${r}_{3}=(7.0-{I}_{i})/4.0 {I}_{i}>3.0$$

$${r}_{4}=({I}_{i}+8.5)/12.5 {I}_{i}\le 4.0$$

$${r}_{4}=(8-{I}_{i})/4.0 {I}_{i}>4.0$$

$${r}_{5}=({I}_{i}+7.5)/12.5 {I}_{i}\le 5.0$$

19 $${r}_{5}=1.0 {I}_{i}>5.0$$

The membership degree *r*_ij_ was calculated through the above membership function, and then the membership degree vector was obtained: *R*_*iz*_ = (${r}_{i1}$*,*…*,*${r}_{im}$), $\sum_{j=1}^{m}{r}_{ij}=1$. In this model, the number of comments was 5, there was: *m* = 5, *R*_*i5*_ = (${r}_{i1}$*,*…*,*${r}_{i5}$). The comprehensive evaluation membership judgment matrix *R*=$\left({R}_{i1}, {R}_{i2}, {R}_{i3}, {R}_{i4}\right)$ was obtained through matrix synthesis operation. The R, TVOC, C, and OI were used to represent R-value, the total concentration of VOCs, the concentration of non-assessable compounds, and the total odor intensity. The membership judgment matrix of comprehensive evaluation can be expressed as follows equation:

20 $$R=\left({R}_{i1}, {R}_{i2}, {R}_{i3}, {R}_{i4}\right)=\left(\begin{array}{c}\begin{array}{c}{r}_{11}\\ {r}_{21}\\ \vdots \end{array}\\ {r}_{n1}\end{array}\right.\begin{array}{c}\begin{array}{c}{r}_{12}\\ {r}_{22}\\ \vdots \end{array}\\ {r}_{n2}\end{array}\begin{array}{c}\begin{array}{c}\dots \\ \dots \\ \ddots \end{array}\\ \dots \end{array}\left.\begin{array}{c}\begin{array}{c}{r}_{1m}\\ {r}_{2m}\\ \vdots \end{array}\\ {r}_{nm}\end{array}\right)=\left(\begin{array}{c}\begin{array}{c}{r}_{11}\\ {r}_{21}\\ \vdots \end{array}\\ {r}_{41}\end{array}\right.\begin{array}{c}\begin{array}{c}{r}_{12}\\ {r}_{22}\\ \vdots \end{array}\\ {r}_{42}\end{array}\begin{array}{c}\begin{array}{c}\dots \\ \dots \\ \ddots \end{array}\\ \dots \end{array}\left.\begin{array}{c}\begin{array}{c}{r}_{15}\\ {r}_{25}\\ \vdots \end{array}\\ {r}_{45}\end{array}\right)=\left(\begin{array}{c}\begin{array}{c}{r}_{\mathrm{R },\mathrm{ A}}\\ {r}_{\mathrm{TVOC},\mathrm{A}}\\ {r}_{\mathrm{C},\mathrm{A}}\end{array}\\ {r}_{\mathrm{OI},\mathrm{A}}\end{array}\right.\begin{array}{c}\begin{array}{c}{r}_{\mathrm{R },\mathrm{B}}\\ {r}_{\mathrm{TVOC},\mathrm{B}}\\ {r}_{\mathrm{C},\mathrm{B}}\end{array}\\ {r}_{\mathrm{OI},\mathrm{B}}\end{array}\left.\begin{array}{c}\begin{array}{c}{r}_{\mathrm{R },\mathrm{C}}\\ {r}_{\mathrm{TVOC},\mathrm{C}}\\ {r}_{\mathrm{C},\mathrm{C}}\end{array}\\ {r}_{\mathrm{OI},\mathrm{C}}\end{array}\begin{array}{c}\begin{array}{c}{r}_{\mathrm{R },\mathrm{Q}}\\ {r}_{\mathrm{TVOC},\mathrm{Q}}\\ {r}_{\mathrm{C},\mathrm{Q}}\end{array}\\ {r}_{\mathrm{OI},\mathrm{Q}}\end{array}\begin{array}{c}\begin{array}{c}{r}_{\mathrm{R },\mathrm{N}}\\ {r}_{\mathrm{TVOC},\mathrm{N}}\\ {r}_{\mathrm{C},\mathrm{N}}\end{array}\\ {r}_{\mathrm{OI},\mathrm{N}}\end{array}\right)$$

#### Determination of weighted vector for indicator (*A*)

In this model, to calculate the correlation degree of the impact of different indicators on health, the relevant test data are randomly selected, and the weights of different indicators are comprehensively analyzed by grey relational analysis. The weight analysis indicators in this part include R-value, the total concentration of VOCs, the concentration of non-assessable compounds, and the total odor intensity.

#### Normalized fuzzy comprehensive evaluation vector (${B}^{^{\prime}}$)

The fuzzy comprehensive evaluation vector *B* can be obtained by multiplying the membership matrix R of each index and its weight coefficient (Eq. (21)). Therefore, the comprehensive membership vector is obtained.

21 $$\mathrm{B}={[{b}_{j}]}_{m}={\left[{\sum }_{i=1}^{n}({w}_{i}*{r}_{ij})\right]}_{m}$$

In the equation, *B* is the fuzzy comprehensive evaluation vector, *b*_j_ is the comprehensive membership of the object in *v*_*j,*_, and *r*_*ij*_ is the subordinate degree of the object to *v*_*j*_ for indicator *u*_*i*_.

Through the application of normalized data, the normalized fuzzy comprehensive evaluation vector (${B}^{^{\prime}}$) was gotten, which showed in Eq. (22).

22 $${B}^{\mathrm{^{\prime}}}={{[b}_{j}^{\mathrm{^{\prime}}}]}_{m}={\left[\frac{{b}_{j}}{{\sum }_{j}{b}_{j}}\right]}_{m}.$$

The number in this model is 5, so there is m = 5.

The final evaluation grade is gotten based on the principle of maximum membership, and combined with the control item, according to AgBB and relevant standards.

#### Calculation of the fuzzy comprehensive index (*P*)

The health grade can be evaluated by the following model. However, people often need to compare the products with the same health grade in daily life. Therefore, the normalized fuzzy comprehensive evaluation vector ${B}^{^{\prime}}$ is processed, and the common weighted average principle of fuzzy comprehensive evaluation vector is used to convert the comprehensive score of the results. The different grades were regarded as a relative unknown and had continuity. Referring to the standard comprehensive evaluation method of public place health, the rank of 0.01, 0.25, 0.5, 0.75, and 1 was taken for each grade. The fuzzy comprehensive index (*P*), reflecting the relative position of the evaluated product was calculated by weighted summation of the rank of each level in vector ${B}^{^{\prime}}$(Eq. (23)). The smaller the fuzzy comprehensive index (P), the higher the health level of the product, on the contrary, the lower the health level. Considering the limit of the control item, the horizontal comparison of health between different multi-component compound release materials is only for the materials with the same health grade.

23 $$P=\frac{{\sum }_{j=1}^{n}{b}_{j}^{k}\cdot m}{{\sum }_{j=1}^{n}{b}_{j}^{k}}$$

### Programmed of computer software

To realize the rapid calculation of the above multi-component compound comprehensive evaluation model, the Computer Software “GRA–FCE multi-component release material health grade comprehensive evaluation software V1.0”, which has obtained the copyright registration certificate of Computer Software from the State Copyright Administration of the People’s Republic of China (Registration No: 2021SR0379871) was programmed by Qt computer technology. Through the software, the procedure of products health grade evaluation and the comparison of products under the same health grade can be conducted.

### Practical examples

The practical examples were conducted on the basis of related research results^15^^.^ The related index value of *Choerospondias axillaris* with different lacquers after 28 days was shown in Table 2. The Comprehensive evaluation model for health grade was used to evaluate the health grade of *Choerospondias axillaris* samples. The steps were as follows:

**Table 2:** Index value of *Choerospondias axillaris* (Roxb.) Burtt et Hill with different lacquers^7^.

Products	R	TVOC (µg/m^3^)	The concentration of Non-assessable compounds (µg/m^3^) (Sum of VOC and VVOC with unknown LCI)	Total odor intensity
UV	2.42	488.89	89.03	25.8
PU	1.52	1072.27	27.76	34.8
Waterborne coating	0.33	593.17	449.44	24.0

#### Determination of the indicator set (*U*)

24 $$U=\left\{{u}_{i}\right\}=\left\{{u}_{1},{u}_{2},{u}_{3},{u}_{4}\right\}=\left\{R ,\mathrm{TVOC},{\sum \mathrm{C}}_{\mathrm{non}-\mathrm{assessable}},\mathrm{ total odor intensity}\right\}$$

#### Establishment of comment set (*V*)

25 $$V=\left\{{v}_{i}\right\}=\left\{{v}_{1},{v}_{2},{v}_{3},{v}_{4},{v}_{5}\right\}=\left\{\mathrm{A},\mathrm{B},\mathrm{C},\mathrm{Q},\mathrm{ N}\right\}$$

#### Calculation of quality index (*I*_*i*_)

The quality index of *Choerospondias axillaris* with different lacquers was calculated by the Eq. (17), which showed in Table 3.

**Table 3:** Quality index of *Choerospondias axillaris* (Roxb.) Burtt et Hill with different lacquers.

Products	R	TVOC (µg/m^3^)	The concentration of Non-assessable compounds (µg/m^3^) (Sum of VOC and VVOC with unknown LCI)	Total odor intensity
UV	4.1578	1.9603	3.5612	4.0107
PU	4.0578	4.0080	1.1104	4.1307
Waterborne coating	1.3200	2.3727	4.0353	3.8000

#### Establishment of membership judgment matrix (*R*)

The membership *r*_ij_ was gotten by bringing the above quality index (*I*_*i*_) into the corresponding membership function with Eq. (19).

For the R-value of *Choerospondias axillaris* with UV lacquer, the membership function was gotten by the calculation, there is

$${r}_{1}=(5-4.1578)/4.0=0.2106$$

$${r}_{2}=(6-4.1578)/4.0=0.4606$$

26 $${r}_{3}=(7-4.1578)/4.0=0.7106$$

$${r}_{4}=(8-4.1578)/4.0=0.9606$$

$${r}_{5}=(4.1578+7.5)/12.5=0.9326$$

So, there is *R*_*i*1_ = (0.2106, 0.4606, 0.7106, 0.9606, 0.9326).

Similarly, the membership function of TVOC, the concentration of non-assessable compounds, and total odor intensity were gotten as follows:

$$R_{i2} \, = \,\left( {0.{7599}, \, 0.{9968}, \, 0.{9168}, \, 0.{8368}, \, 0.{7568}} \right);$$

27 $$R_{i3} \, = \,\left( {0.3597,\,0.6097,\,0.8597,\,0.9649,\,0.8849} \right)$$

$$R_{i4} \, = \,\left( {0.{2473}, \, 0.{4973}, \, 0.{7473}, \, 0.{9973}, \, 0.{92}0{9}} \right).$$

Based on the membership vector *R*_*iz*_, the membership judgment matrix *R*_*UV*_ for comprehensive evaluation of *Choerospondias axillaris* with UV lacquer was obtained as follows.

28 $${R}_{\text{UV}}=\left({R}_{i1},{R}_{i2},{R}_{i3},{R}_{i4}\right)=\left(\begin{array}{c}\begin{array}{c}0.2106\\ 0.7599\\ 0.3597\end{array}\\ 0.2473\end{array}\right.\hspace{0.33em}\hspace{0.33em}\hspace{0.33em}\begin{array}{c}\begin{array}{c}0.4606\\ 0.9968\\ 0.6097\end{array}\\ 0.4973\end{array}\left.\hspace{0.33em}\hspace{0.33em}\hspace{0.33em}\begin{array}{c}\begin{array}{c}0.7106\\ 0.9168\\ 0.8597\end{array}\\ 0.7473\end{array}\hspace{0.33em}\hspace{0.33em}\hspace{0.33em}\begin{array}{c}\begin{array}{c}0.9606\\ 0.8368\\ 0.9649\end{array}\\ 0.9973\end{array}\hspace{0.33em}\hspace{0.33em}\hspace{0.33em}\begin{array}{c}\begin{array}{c}0.9326\\ 0.7568\\ 0.8849\end{array}\\ 0.9209\end{array}\right)$$

The membership judgment matrix of *R*_*PU*_ and *R *_*Waterborne coating*_ was gotten on the same principle, there was:

29 $${R}_{\text{PU}}=\left({R}_{i1},{R}_{i2},{R}_{i3},{R}_{i4}\right)=\left(\begin{array}{c}\begin{array}{c}0.2356\\ 0.2480\\ 0.9724\end{array}\\ 0.2173\end{array}\right.\hspace{0.33em}\hspace{0.33em}\hspace{0.33em}\begin{array}{c}\begin{array}{c}0.4856\\ 0.4980\\ 0.9288\end{array}\\ 0.4673\end{array}\left.\hspace{0.33em}\hspace{0.33em}\hspace{0.33em}\begin{array}{c}\begin{array}{c}0.7356\\ 0.7480\\ 0.8488\end{array}\\ 0.7173\end{array}\hspace{0.33em}\hspace{0.33em}\hspace{0.33em}\begin{array}{c}\begin{array}{c}0.9856\\ 0.9980\\ 0.7688\end{array}\\ 0.9673\end{array}\hspace{0.33em}\hspace{0.33em}\hspace{0.33em}\begin{array}{c}\begin{array}{c}0.9246\\ 0.9206\\ 0.6888\end{array}\\ 0.9305\end{array}\right)$$

30 $${R}_{Waterborne coating}=\left({R}_{i1},{R}_{i2},{R}_{i3},{R}_{i4}\right)=\left(\begin{array}{c}\begin{array}{c}0.9200\\ 0.6568\\ 0.2412\end{array}\\ 0.3000\end{array}\right.\hspace{0.33em}\hspace{0.33em}\hspace{0.33em}\begin{array}{c}\begin{array}{c}0.9456\\ 0.9068\\ 0.4912\end{array}\\ 0.5500\end{array}\hspace{0.33em}\hspace{0.33em}\hspace{0.33em}\left.\begin{array}{c}\begin{array}{c}0.8656\\ 0.9498\\ 0.7412\end{array}\\ 0.8000\end{array}\hspace{0.33em}\hspace{0.33em}\hspace{0.33em}\begin{array}{c}\begin{array}{c}0.7856\\ 0.8698\\ 0.9912\end{array}\\ 0.9840\end{array}\hspace{0.33em}\hspace{0.33em}\hspace{0.33em}\begin{array}{c}\begin{array}{c}0.7056\\ 0.7898\\ 0.9228\end{array}\\ 0.9040\end{array}\right)$$

#### Normalized fuzzy comprehensive evaluation vector (${B}^{^{\prime}}$)

Due to the weighted vector for indicators has been gotten, the fuzzy comprehensive evaluation vector *B*_UV_, *B*_PU_, *B*_waterborne coatings_ of *Choerospondias axillaris* with UV, PU, and waterborne coatings after 28 days were calculated by multiplying the membership matrix *R* of each index and its weight coefficient, there was:

31 $${\text{B}}_{{{\text{UV}}}} \,{ = }\,\left( {{0}{\text{.3975, 0}}{.6440, 0}{\text{.8061, 0}}{.9374, 0}{\text{.8728}}} \right){ }$$

32 $$B_{PU} \, = \,\left( {0.3908, \, 0.5785, \, 0.7585, \, 0.9385, \, 0.8749} \right)$$

33 $${\text{B}}_{{\text{waterborne\, coatings}}} \,{ = }\,\left( {{0}{\text{.5556, 0}}{.7429, 0}{\text{.8457, 0}}{.8998, 0}{\text{.8222}}} \right)$$

After the application of normalized data, the normalized fuzzy comprehensive evaluation vector (${B}^{^{\prime}}$) was gotten as follows:

34 $${B}_{UV}^{^{\prime}}= 0.1087, 0.1761, 0.2204, 0.2563, 0.2386$$

35 $${B}_{PU}^{^{\prime}}= 0.1104, 0.1634, 0.2142, 0.2650, 0.2471$$

36 $${B}_{\mathrm{waterborne\, coatings }}^{^{\prime}}= 0.1437, 0.1922, 0.2187, 0.2327, 0.2127$$

Based on the principle of maximum membership, it was found that *Choerospondias axillaris* with UV, PU, and waterborne coatings had the highest membership in the grade “Q”. However, according to the limit value for qualified products, It was found that the specific single index of *Choerospondias axillaris* with different lacquers was greater than the limit. The *R* of the UV lacquers sample > 1, the *R* of the PU lacquers sample > 1, and TVOC > 1000 μg⋅m^−3^, and the concentration of non-assessable compounds of waterborne coatings > 100 μg⋅m^−3^. Therefore, the health grade above “Q” is not considered, and the final evaluation of the health grade of the three samples was “N”.

#### Calculation of the fuzzy comprehensive index (*P*)

For the three samples that had the same health grade, the fuzzy comprehensive index was calculated through the Eq. (23). The *P*_UV_, *P*_PU_, and *P*_waterborne coatings_ were 0.5861, 0.5949, and 0.5461 respectively, which showing among the three materials with “N” health grade, the health grade of waterborne coatings was relatively the best, followed by the UV. The health grade of PU was the worst. The result agreed with the research study of Wang et al*.* However, the results of this study further prove that the health grade of UV was better than PU.

## Results and discussion

### Characterization of odor‑active substances of Xylosma racemosum (Sieb. et Zucc.) Miq. with different lac‑quer treatments

To evaluate the health grade of *Xylosma racemosum* (Sieb. et Zucc.) Miq. with different lacquer treatments, the release characteristic was studied first. The emission of *Xylosma racemosum* (Sieb. et Zucc.) Miq. with different lacquer treatments was explored by the technology of GC–MS–O. Totally 21 kinds of odorants were identified from three samples, including aromatics (4 substances), aldehydes and ketones (1 substance), esters (9 substances), and alcohols (6 substances), and others (1 substance) (Table 4). It was found that the odor intensity of odorants from samples was generally not high.

**Table 4:** Identification of odor–active compounds of *Xylosma racemosum* (Sieb. et Zucc.) Miq. with different lacquer.

NO	Formula	Compounds	Odor character	Odor intensity			
				None	PU	UV	Water
1	C_9_H_12_	1,2,3–trimethyl–benzene	Spearmint-like	0	1.0	0	0
2	C_8_H_10_	1,3-dimethyl-benzene	Metality	0	1.5	0.6	0
3	C_4_H_10_O	1–butanol	alcohol-like	0	1.0	0	0
4	C_4_H_10_O_2_	1–methoxy–2–propanol	Pleasant	0	0.6	0	0
5	C_6_H_12_O_3_	1–methoxy–2–propyl acetate	Sweety	0	0.8	0	0
6	C_7_H_16_O_3_	2–(2–methoxypropoxy)–1–propanol	Unpleasant	0	0	0	0.5
7	C_6_H_14_O_3_	2–(2–hydroxypropoxy)–1–propanol	Tobacco-like	0	0	0	2.5
8	C_16_H_30_O_4_	2–methyl–propionicacid,1–(1,1–dimethylethyl)–2–methyl–1,3–propyleneglycolester	disinfectant	0	2.0	0	3.1
9	C_5_H_8_O_2_	2–methyl–2–methyl acrylate	Pungent/fruity	0.5	0	0	0
10	C_7_H_14_O_2_	2–pentanol,acetate	Flowery	0	0.6	0	0
11	C_8_H_18_O	2–ethyl–1–hexanol	Flowery	1.0	0	1.0	1.0
12	C_2_H_4_O_2_	Acetic acid	Vinegar-like	3.0	2.5	0.8	1.0
13	C_8_H_10_	P-xylene	Aromatic/sweety	1.0	1.0	0.5	1.0
14	C_6_H_12_O	Hexanal	Grassy	0.3	0.3	0	0
15	C_7_H_12_O_4_	Glutaric acid, dimethyl ester	Pleasant	0	1.0	0	0
16	C_8_H_10_	Ethyl benzene	aromatic	0	1.8	0	0
17	C_2_H_6_O	Ethanol	alcohol-like	1.0	0.5	0.8	0.5
18	C_6_H_12_O_2_	Acetic acid, 1-methylpropyl ester	Fruity	0	1.3	0	0
19	C_6_H_12_O_2_	Acetic acid, 2-methylpropyl ester	Fruity	0	0.5	0	0
20	C_6_H_12_O_2_	Acetic acid, butyl ester	Fruity	0	1.2	0	0
21	C_4_H_8_O_2_	Ethyl acetate	Fruity	1.4	1.0	2.8	1.0

**Figure 2:**
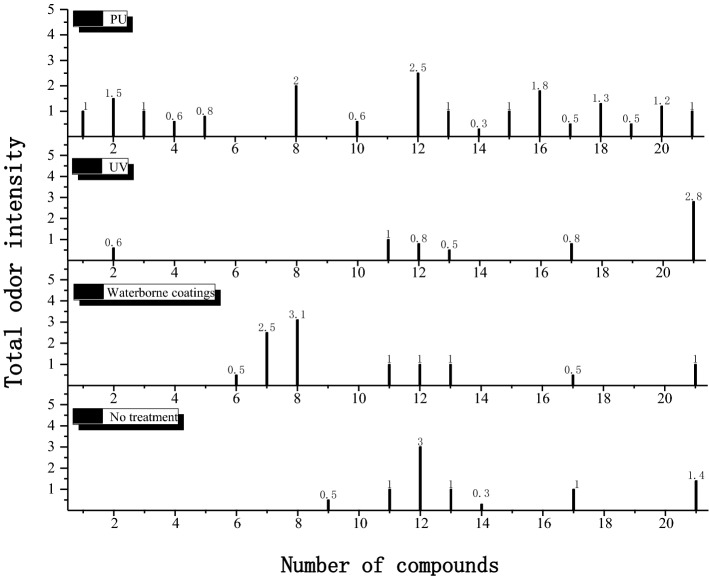
Odor-active compounds of *Xylosma racemosum* (Sieb. et Zucc.) Miq. with different lacquer decorations (The numbers correspond to the numbers provided in Table 4).

The comparison of odor-active compounds was shown in Fig. 2. It was found that the treatment of PU, UV, and water-based coatings have a certain sealing effect on some odor-active compounds from wood. After lacquer treatment, the odor intensity of three odorants, namely, 2-methyl-2-methyl acrylate (No. 9), acetic acid (No. 12), and ethanol (No. 17), decreased. The intensity of 2-methyl-2-methyl acrylate decreased to zero after treatment. At the same time, PU coating and waterborne coating inhibited the release of ethyl acetate (No. 21), UV coating and waterborne coating inhibited the release of hexanal (No. 14), PU coating and UV coating inhibited the release of 2-ethyl-1-hexanol (No. 11) and p-xylene (No. 13), respectively. Some new odorants appeared during the process at the same time. 12 kinds of odorants, including 1,2,3–trimethyl–benzene (No. 1), 1,3–dimethyl–benzene (No. 2), 1–methoxy–2–propyl acetate (No. 5), 2–methyl–propionic acid, 1–(1,1–dimethyl ethyl)–2–methyl–1,3–propylene glycol ester (No. 8), 1–butanol (No. 3), 1 –methoxy –2–propanol (No. 4), 2–pentanol, acetate (No. 10), Glutaric acid, dimethyl ester (No. 15), ethyl benzene (No. 16), acetic acid, 1-methyl propyl ester (No. 18), acetic acid, 2-methyl propyl ester (No. 19) and acetic acid, butyl ester (No. 20) arose after PU lacquer. The odor intensity increased from 0 to 1.0, 1.5, 0.8, 2.0, 1.0, 0.6, 0.6, 1.0, 1.8, 1.3, 0.5, and 1.2, respectively. The waterborne coating board increased the release of three odor-active compounds: 2–(2–methoxypropoxy)–1–propanol (No. 6), 2–(2–hydroxypropoxy)–1–propanol (No. 7), and 2–methyl–propionic acid, 1–(1,1–dimethyl ethyl)–2–methyl–1,3–propylene glycol ester (No. 8), and the odor intensity increases from 0 to 0.5, 2.5 and 3.1 respectively. The odor intensity of 1,3-dimethyl-benzene (No. 2) and ethyl acetate (No. 21) increased by 0.6 and 1.4 respectively after UV coating.

### VOCs/VVOCs and odors emission properties of *Xylosma racemosum (Sieb. et Zucc.) Miq.* with different lacquer treatments

**Figure 3:**
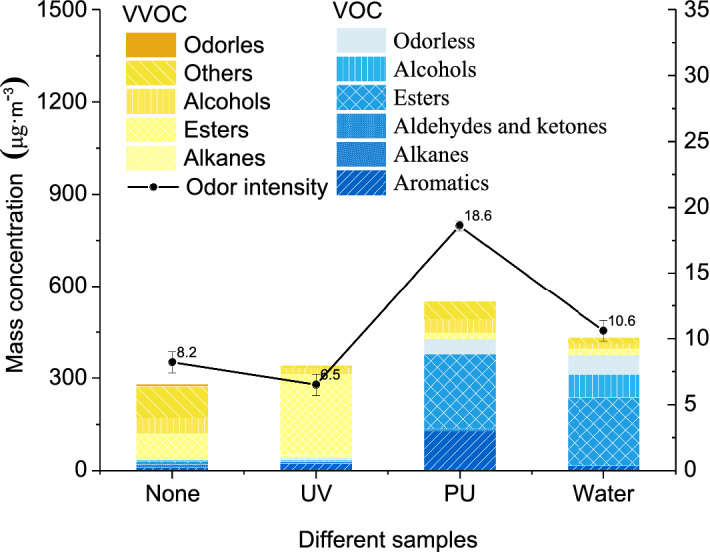
Concentrations of VOC/VVOC components and total odor intensity from *Xylosma racemosum* (Sieb. et Zucc.) Miq. with different lacquer decorations.

The concentrations of VOCs/VVOCs components and total odor intensity from *Xylosma racemosum* (Sieb. et Zucc.) Miq. with different lacquer decorations was shown in Fig. 3. It was found that the total mass concentration of VOCs/VVOCs of three kinds of lacquered wood was higher than untreated wood (279.97 μg m^−3^). The mass concentration of PU lacquered wood was the highest (550.41 μg m^−3^), followed by waterborne coating (432.53 μg m^−3^), and UV coating (341.36 μg m^−3^). VOCs component was the main release component of PU and waterborne coating board, accounting for 77.46% and 86.64% of the total concentration respectively. The odor components of PU coating mainly come from esters and aromatics in VOCs components, and a small number of aldehydes and ketones were distributed in VOCs. VVOCs components were distributed in alcohols, esters, and others. The odor components of waterborne coatings wood mainly came from the esters and alcohols in VOCs components. A small number of aromatics was distributed in VOCs and esters, alcohols, and other compounds in VVOCs. The main odor components of wood without treatment and UV coating wood are VVOCs, accounting for 88.06% and 87.52% of the total concentration respectively. Among them, the VVOCs components of others, esters, and alcohols were the main release of untreated wood. A small amount of VOCs odor released was distributed in aromatics, alkanes, aldehydes and ketones, esters, and alcohols. The main odor source of UV lacquered wood was esters, and a small amount of alcohols, alkanes, and others. The release of ester VVOCs of UV lacquered wood was mainly from ethyl acetate with a concentration of 260.13 µg/m^3^, which accounted for 76.20% of the total concentration. A similar phenomenon had also been found in related studies^15^. It was analyzed that the ethyl acetate may come from the solvent of UV coating, the wood itself, and the product of the interaction between the compounds in the solvent and the compounds in the wood, which should be given close attention for its high concentration.

UV had an inhibitory effect on alcohol and other compounds. The concentration decreased by 31.44 μg m^−3^and 95.58 μg m^−3^ respectively. However, at the same time, it increased the release of esters, and the concentration increased by 177.29 μg m^−3^. PU increased the release of aromatics and esters, and the concentration increased by 117.68 μg m^−3^ and 239.39 μg m^−3^, but it had a certain inhibitory effect on the release of esters and other compounds. The water-based coating increased the release of esters and alcohols odor compounds of oak VOCs components, and the concentrations increased by 217.17 μg m^−3^ and 70.50 μg m^−3^, but it showed it inhibit the release of esters, alcohols and other compounds in the odorous components. After UV coating treatment, the total odor intensity of the board decreased. After being treated with PU and waterborne coating, the total odor intensity of the plate increased. The total odor intensity of wood with UV coating was the lowest (6.5), followed by untreated wood (8.2), water-based coating wood (10.6), and PU-coating wood (18.6).

### Evaluation of the material health grade

The index value was obtained from the above research. The three samples were then evaluated by the comprehensive evaluation model. The fast computing was realized by the Computer Software of “GRA – FCE multi-component release material health grade Comprehensive Evaluation Software V1.0”. The data and the output were showed in Table 5. It was shown that the UV lacquered *Xylosma racemosum* (Sieb. et Zucc.) Miq. was with the best health grade of “A”. The health grade of PU and waterborne coatings boards was “N”. The main reason for the health grade of PU and waterborne coatings boards was the concentration of non-assessable compounds > 100 μg m^−3^. For there were two samples that had the same health grade of “N”, the fuzzy comprehensive index *P* was analyzed through the Computer Software. The outputs of *P*_PU_ and *P*_waterborne coatings_ were 0.5151and 0.4950, showing the health grade of the water-based coating was higher than that of PU. The final evaluation result of *Xylosma racemosum* (Sieb. et Zucc.) Miq. with different lacquer decorations was: UV > water-based coating > PU.

**Table 5:** Index value and result output of *Xylosma racemosum* (Sieb. et Zucc.) Miq. decorated with different lacquer decorations.

Samples	R	TVOC (µg/m^3^)	The concentration of Non-assessable compounds (µg/m^3^) (Sum of VOC and VVOC with unknown LCI)	Total odor intensity	Output
UV	0.1167	42.60	42.82	6.50	A
PU	0.1861	426.33	299.71	18.60	N
Waterborne coating	0.0748	374.73	310.61	10.60	N

## Conclusion

To guide the selection of a suitable alternative material that considers multiple factors (R-value, total VOC concentration, concentration of non-assessable compounds, and total odor intensity), a comprehensive evaluation model of health grade of multi-component compound release materials was proposed based on the mathematical methods of GRA and FCE in this paper. A health evaluation procedure scheme of the AgBB as well as standards of the forestry industry of the People's Republic of China and standards of the health industry of the People’s Republic of China were referred to during the process. In order to facilitate the calculation of compound comprehensive evaluation models, a computer software has been programmed using computer technology of Qt. The emission and odor characteristics of *Xylosma racemosum* (Sieb. et Zucc.) Miq. with PU, UV, and waterborne coating were investigated with the technology of GC–MS/O for further investigation.

Based on the principle of maximum membership and combined with the limit value of related standards, it found that, for *Choerospondias axillaris* samples, the health grade of waterborne coatings was relatively the best, followed by the UV, the health grade of PU was the worst (The fuzzy comprehensive index *P* were: *P*_UV_ = 0.5861, *P*_PU_ = 0.5949, *P*_waterborne coatings=_0.5461). For *Xylosma racemosum* (Sieb. et Zucc.) Miq. samples, the health grade of UV coating was “A”, and the boards with PU and the waterborne coating were “N”. The fuzzy comprehensive index were: *P*_PU_ = 0.5151, and *P*_waterborne coatings_ = 0.4950_,_ which showed the health grade of the three samples was: UV > waterborne coating > PU.

The results of this study provide a platform for the comprehensive evaluation of the board’s health grade. In the meantime, this study provides guidance to consumers on how to use products sensibly and prevent indoor air pollution. The future work will involve further optimization and verification with other models. Additionally, given the subjective nature of odor quantification, ongoing efforts should be made to improve the accuracy of odor intensity calculations.

## Methods

### Materials

The samples of *Xylosma racemosum* (Sieb. et Zucc.) Miq. with different lacquers were used in this experiment for further validation model. Samples of wood were obtained from Senbao wood industry (Shenyang City, Liaoning, China). A round piece (60 mm diameter, 16 mm thickness) was cut from the sample, with an exposed surface area of 5.65 × 10^−3^ m^2^. The wood samples were dried to an equilibrium moisture content (10 × 2%) before being coated with PU, UV-cured coatings, and waterborne coatings. Finishing parameters were determined by reference to related research^15^.

### Sampling and analytical methodology

After 28 days of lacquered treatment, 2 L of emission, including VOCs and VVOCs, was adsorbed using a microchamber/thermal extractor with two types of tubes of Tenax-TA tube and tubes with multi-sorbents of carbopack C, carbopack B, and carboxen 1000 (Markes International, South Wales, UK). The cell volume was 1.35 × 10^−4^ m^3^, and the loading rate (the ratio of the panel area to the microchamber volume) was 41.85 m^2^/m^3^. The environment conditions were as follows: temperature, 23 ± 2 °C; relative humidity, 40 ± 10%; ratio of air exchange rate to loading factor, 0.5 m^3^ m^−2^ h^−1^. Purified nitrogen was supplied throughout the experiment.

The method of combination with human and apparatus was used in this experiment. TD-GC–MS/O was used to analyze the emission, which was matched with MS spectra from the National Institute of Standards and Technology (NIST) and Wiley MS libraries. The DSQ II series quadrupole gas chromatography–mass spectroscopy (GC–MS) unit came from Thermo Fisher Scientific (Schwerte, Germany). Chromatography was performed with a DB-5 quartz capillary column (30,000 m long × 0.26 mm inner diameter × 0.25 µm particle size; Agilent Technologies, Santa Clara, CA). The parameters were as follows: cold-trap adsorption temperature, − 15 °C; thermal desorption temperature, 280 °C; thermal analysis time, 10 min; injection time, 1 min. Helium was used as the carrier gas. The chromatographic column was initially kept at 40 °C for 2 min, and then the temperature was increased to 50 °C (in 2 °C min^−1^ increments) and held at that temperature for 4 min. Finally, the temperature was increased to 250 °C in 10 °C min^−1^ increments and held for 8 min, with the injection port temperature also at 250 °C. The parameters of GC–MS conditions were as follows: ionization mode, electron ionization; ion energy, 70 eV; ion source temperature, 230 °C; transmission line temperature, 270 °C; mass scan range, 50–650 atomic mass units. The Sniffer 9100 Olfactory Detector came from Brechbühler (Echallens, Switzerland). The transmission line temperature was 150 °C, and nitrogen was used as the carrier gas through a purge valve. Moist air was added to prevent dehydration of the nasal mucosa of the odor assessors. Direct intensity methods were used to analyze the compounds. Compounds with matching degrees of 800 or more were retained. The quantitative method referred to the Chinese national standard GB/T 29899-2013^35^.

The part of odor determination was based on specific screening and training recommendations in ISO12219-7. The experimental environment was set to National Standards Authority of Ireland reference standard EN 13725-2003. Four assessors (between 20 and 30 years old, with no history of smoking and no olfactory organ disease) were chosen to form an odor-analysis evaluation group. The odorants were identified based on their odor qualities as perceived at the sniffing port, their retention indices on the DB-5, calculated on the basis of a series of homologous alkanes (C_6_–C_30_) according to Van den Dool and Kratz^36^, their mass spectra, and compared with the literature. A six-point scale ranging from 0 to 5 was used for intensity judgment according to Japanese Ministry of the Environments standards: 0 = none, 1 = very weak, 2 = weak, 3 = moderate, 4 = strong, and 5 = very strong. The fingerprint span method was used simultaneously to verify the results.

### Ethical statement

The study was conducted in agreement with the Declaration of Helsinki. The research contents and methods as mentioned above were evaluated and approved by College of Materials Science and Engineering (Ethics committee), Northeast Forestry University. Informed consent was obtained from all subjects participating in the study.
